# Supplementary material for: Is Standardization Necessary for Sharing of a Large Mid-Infrared Soil Spectral Library?
Source: Sensors (Basel). 2020 Nov 25;20(23):6729. doi: 10.3390/s20236729 (PMC7728050; doi:10.3390/s20236729)
Supplement: Supplementary file 1 [file sensors-20-06729-s001.pdf]

Is standardization necessary for sharing of a large mid-infrared soil spectral library?

Shree R.S. Dangal and Jonathan Sanderman

Woodwell Climate Research Center, Falmouth MA-02540, United States

Corresponding Authors:

E-mail: sdangal@woodwellclimate.org (S. Dangal)

jsanderman@woodwellclimate.org (J. Sanderman)

Contents of this file

Tables S1-S9

Figures S1-S4

Table S1. Performance of predictive models using baseline offset and first derivative transformation for all soil properties

Models	Data	Properties	N	Baseline Offset			First Derivative		
				Bias	R^2	RMSE	Bias	R^2	RMSE
PLSR	KSSL	BD	13667	0.01	0.82	0.19	0.01	0.83	0.18
		$CaCO_3$	9365	0.06	0.98	1.84	0.05	0.98	1.61
		Clay	37187	0.30	0.89	5.36	0.26	0.90	4.90
		OC	55598	0.04	0.99	1.61	0.04	0.99	1.54
		pH	39347	0.01	0.81	0.57	0.01	0.82	0.55
Cubist	KSSL	BD	13667	0.00	0.89	0.15	0.00	0.87	0.16
		$CaCO_3$	9365	0.01	0.99	0.96	0.02	0.99	1.04
		Clay	37187	0.10	0.96	3.06	0.07	0.96	3.30
		OC	55598	0.01	0.99	0.90	0.00	0.99	0.90
		pH	39347	0.00	0.95	0.30	0.01	0.92	0.37

Table S2. Performance of NSSC-KSSL predictive models on transfer spectra (Set I) of the USA soil samples comprising of different combinations of spectra and models (KSSL, Woodwell, $Woodwell_{PDS}$ and $Woodwell_{DC}$) using the baseline offset transformation.

		MBL				PLSR				Cubist			
	Spectra	Bias	R^2	RMSE	RPIQ	Bias	R^2	RMSE	RPIQ	Bias	R^2	RMSE	RPIQ
BD (n = 108)	KSSL	0.4	0.51	0.47	0.64	0.5	0.37	0.52	0.58	0.4	0.55	0.46	0.66
	Woodwell	0.5	0.28	0.54	0.56	0.5	0.40	0.58	0.52	0.5	0.21	0.6	0.50
	$Woodwell_{PDS}$	0.4	0.45	0.50	0.61	0.5	0.35	0.53	0.57	0.4	0.27	0.55	0.55
	$Woodwell_{DC}$	0.0	0.32	0.22	1.38	0.0	0.17	0.24	1.26	0.0	0.03	0.46	0.66
$CaCO_3$ (n=212)	KSSL	0.0	0.97	3.15	4.77	0.0	0.95	3.98	3.78	-0.2	0.98	2.66	5.65
	Woodwell	0.6	0.97	3.58	4.20	1.9	0.96	5.02	3.00	-1.0	0.88	7.49	2.01
	$Woodwell_{PDS}$	0.8	0.98	3.10	4.85	1.6	0.96	4.62	3.25	0.0	0.93	5.33	2.82
	$Woodwell_{DC}$	0.1	0.98	2.63	5.72	-0.1	0.89	6.81	2.21	-0.1	0.89	6.34	2.37
Clay (n= 312)	KSSL	0.1	0.96	4.04	7.32	0.9	0.83	7.98	3.70	0.5	0.95	4.29	6.89
	Woodwell	2.4	0.77	9.73	3.04	3.2	0.76	10.38	3.85	-5.4	0.43	16.79	1.76
	$Woodwell_{PDS}$	1.9	0.90	6.73	4.39	1.9	0.77	9.52	3.10	1.9	0.77	9.75	3.03
	$Woodwell_{DC}$	0.0	0.81	8.55	3.46	0.7	0.74	9.99	2.96	0.7	0.76	9.64	3.07
OC (n = 409)	KSSL	0.1	0.99	1.54	3.39	0.1	0.95	2.69	1.94	0.0	0.98	1.53	3.41
	Woodwell	-1.1	0.97	2.53	2.06	-1.1	0.96	2.69	1.94	-1.2	0.95	3.15	1.66
	$Woodwell_{PDS}$	0.4	0.98	1.90	2.75	0.2	0.96	2.56	2.04	0.5	0.97	2.39	2.19
	$Woodwell_{DC}$	0.2	0.96	2.38	2.19	0.1	0.95	2.83	1.85	0.3	0.93	3.37	1.55
pH (n = 331)	KSSL	0.0	0.94	0.36	7.18	0	0.77	0.71	3.64	-0.0	0.89	0.5	5.17
	Woodwell	0.3	0.78	0.76	3.40	0.5	0.70	0.95	2.72	0.6	0.5	1.42	1.82
	$Woodwell_{PDS}$	0.0	0.83	0.61	4.24	-0.1	0.68	0.97	2.66	-0.2	0.66	1.14	2.27
	$Woodwell_{DC}$	-0.1	0.73	0.77	3.36	0.0	0.67	0.85	3.04	-0.0	0.69	0.82	3.15

*The performance of $Woodwell_{DC}$ is based on leave-one-out cross validation. The sample size of each property are different because not all predicted soil properties have measured data to evaluate model performance.

Table S3. Performance of NSSC-KSSL predictive models on transfer spectra (Set I) of the USA soil samples comprising of different combinations of spectra and models (KSSL, Woodwell, $Woodwell_{PDS}$ and $Woodwell_{DC}$) using the first derivative transformation.

		MBL				PLSR				Cubist			
	Spectra	Bias	R^2	RMSE	RPIQ	Bias	R^2	RMSE	RPIQ	Bias	R^2	RMSE	RPIQ
BD	KSSL	0.4	0.57	0.45	0.67	0.4	0.36	0.50	0.61	0.4	0.44	0.48	0.63
(n = 108)	Woodwell	0.4	0.39	0.50	0.61	0.5	0.31	0.58	0.52	-0.2	0.09	0.73	0.41
	$Woodwell_{PDS}$	0.5	0.46	0.50	0.61	0.4	0.34	0.51	0.59	0.5	0.11	0.68	0.45
	$Woodwell_{DC}$	0.0	0.45	0.20	1.51	0.0	0.36	0.21	1.44	-0.0	0.14	0.26	1.25
$CaCO_3$	KSSL	0.0	0.98	2.53	5.94	-0.1	0.95	4.05	3.71	0.0	0.97	3.06	4.91
(n=212)	Woodwell	0.9	0.97	3.47	4.33	1.6	0.96	4.45	3.38	2.8	0.94	5.91	2.54
	$Woodwell_{PDS}$	0.7	0.97	3.30	4.56	0.9	0.97	3.69	4.07	0.9	0.91	5.57	2.70
	$Woodwell_{DC}$	0.1	0.98	2.30	6.54	-0.1	0.92	5.87	2.56	0.6	0.87	7.11	2.23
Clay	KSSL	0.1	0.97	3.20	9.24	0.6	0.85	7.66	3.86	0.5	0.94	4.91	6.02
(n= 312)	Woodwell	2.5	0.90	6.83	4.33	2.2	0.65	11.68	2.53	-18.0	0.08	39.6	0.75
	$Woodwell_{PDS}$	2.2	0.90	6.84	4.32	3.4	0.72	10.84	2.73	0.4	0.33	23.22	1.27
	$Woodwell_{DC}$	0.5	0.77	9.34	3.16	0.7	0.75	9.71	3.04	0.8	0.68	11.06	2.71
OC	KSSL	0.1	0.98	1.67	3.13	0.2	0.96	2.62	1.99	0.2	0.98	1.67	3.13
(n = 409)	Woodwell	-0.1	0.97	2.11	2.48	-0.7	0.96	2.56	2.04	-0.1	0.89	4.17	1.25
	$Woodwell_{PDS}$	0.4	0.98	2.01	2.60	0.3	0.96	2.57	2.03	0.2	0.7	7.22	0.72
	$Woodwell_{DC}$	0.1	0.96	2.41	2.17	0.1	0.94	3.06	1.71	0.2	0.92	3.61	1.45
pH	KSSL	0.0	0.96	0.28	9.23	0.1	0.78	0.70	3.69	0.0	0.91	0.44	5.88
(n = 331)	Woodwell	0.1	0.75	0.76	3.40	0.2	0.70	0.85	3.04	0.8	0.39	2.51	1.03
	$Woodwell_{PDS}$	0.0	0.83	0.61	4.24	0.1	0.71	0.80	3.23	-0.8	0.1	2.7	0.96
	$Woodwell_{DC}$	-0.1	0.64	0.91	2.84	0.0	0.60	0.94	2.75	0.04	0.57	0.97	2.69

*The performance of $Woodwell_{DC}$ is based on leave-one-out cross validation. The sample size of each property are different because not all predicted soil properties have measured data to evaluate model performance.

Table S4. Performance of NSSC-KSSL predictive models on independent test set (Set II) of the USA soil samples comprising of different combinations of spectra and models (KSSL, Woodwell, $Woodwell_{PDS}$ and $Woodwell_{DC}$) using the baseline offset transformation.

	Data	MBL				PLSR				Cubist			
		Bias	R^2	RMSE	RPIQ	Bias	R^2	RMSE	RPIQ	Bias	R^2	RMSE	RPIQ
BD	KSSL	-0.02	0.71	0.22	2.16	-0.01	0.63	0.26	1.83	-0.04	0.61	0.27	1.76
(n= 290)	Woodwell	-0.07	0.59	0.30	1.59	0.04	0.57	0.28	1.70	-0.02	0.29	0.47	1.01
	$Woodwell_{PDS}$	-0.05	0.64	0.26	1.83	0.01	0.61	0.26	1.83	-0.06	0.51	0.33	1.44
	$Woodwell_{DC}$	0.01	0.70	0.23	2.07	0.02	0.63	0.25	1.90	0.00	0.63	0.26	1.83
clay	KSSL	-0.98	0.93	3.79	5.37	-0.89	0.91	4.42	4.60	-3.68	0.84	7.41	2.75
(n= 286)	Woodwell	-3.30	0.78	7.47	2.72	-1.12	0.84	5.85	3.48	-8.05	0.43	15.14	1.34
	$Woodwell_{PDS}$	-3.21	0.85	6.34	3.21	-3.83	0.84	7.01	2.90	-2.53	0.74	8.62	2.36
	$Woodwell_{DC}$	-0.01	0.89	4.64	4.39	0.22	0.87	5.17	3.94	0.43	0.86	5.30	3.84
OC	KSSL	0.03	0.99	0.70	2.72	0.01	0.99	1.07	1.78	-0.16	0.95	2.00	0.95
(n= 296)	Woodwell	-0.84	0.96	2.20	0.87	-1.34	0.97	2.15	0.89	-0.85	0.97	1.71	1.12
	$Woodwell_{PDS}$	0.03	0.98	1.53	1.25	-0.35	0.98	1.44	1.32	0.02	0.96	1.72	1.11
	$Woodwell_{DC}$	0.08	0.98	1.22	1.56	0.04	0.97	1.57	1.21	0.13	0.97	1.53	1.25
pH	KSSL	0.07	0.95	0.33	7.77	0.12	0.86	0.54	4.75	0.03	0.91	0.48	5.34
(n= 296)	Woodwell	0.42	0.77	0.8	3.21	0.47	0.85	0.72	3.56	0.91	0.61	1.37	1.87
	$Woodwell_{PDS}$	-0.09	0.81	0.62	4.14	0.03	0.84	0.67	3.83	0.05	0.73	0.89	2.88
	$Woodwell_{DC}$	0.03	0.91	0.41	6.26	-0.01	0.90	0.45	5.70	0.03	0.91	0.43	5.97

*The performance of $Woodwell_{DC}$ is based on leave-one-out cross validation. The sample size of each property are different because not all predicted soil properties have measured data to evaluate model performance.

Table S5. Performance of NSSC-KSSL predictive models on independent test set (Set II) of the USA soil samples comprising of different combinations of spectra and models (KSSL, Woodwell, $Woodwell_{PDS}$ and $Woodwell_{DC}$) using the first derivative transformation.

	Data	MBL				PLSR				Cubist			
		Bias	R^2	RMSE	RPIQ	Bias	R^2	RMSE	RPIQ	Bias	R^2	RMSE	RPIQ
BD	KSSL	-0.03	0.71	0.23	2.07	-0.03	0.65	0.25	1.90	0.18	0.07	0.76	0.63
(n= 290)	Woodwell	-0.02	0.55	0.30	1.59	0.05	0.54	0.29	1.64	-0.44	0.24	0.81	0.59
	$Woodwell_{PDS}$	-0.02	0.61	0.27	1.76	-0.04	0.58	0.28	1.70	0.18	0.20	0.52	0.91
	$Woodwell_{DC}$	0.01	0.68	0.24	1.98	0.02	0.63	0.26	1.83	0.00	0.64	0.25	1.90
clay	KSSL	-0.71	0.94	3.62	5.62	0.06	0.91	4.25	4.79	-19.82	0.13	41.87	0.49
(n= 286)	Woodwell	-1.38	0.78	6.83	2.98	-1.70	0.69	8.27	2.46	-15.35	0.07	34.32	0.59
	$Woodwell_{PDS}$	-1.15	0.83	6.14	3.31	0.06	0.77	6.84	2.98	-2.16	0.24	18.65	1.09
	$Woodwell_{DC}$	-0.03	0.85	5.46	3.73	0.27	0.82	5.96	3.41	0.23	0.78	6.73	3.02
OC	KSSL	0.01	0.99	0.71	2.69	-0.03	0.99	0.89	2.14	-1.04	0.87	3.69	0.52
(n= 296)	Woodwell	-0.47	0.96	1.84	1.04	-1.09	0.97	1.91	1.00	-0.04	0.89	3.05	0.63
	$Woodwell_{PDS}$	-0.09	0.98	1.26	1.51	-0.05	0.98	1.32	1.44	-0.83	0.81	5.05	0.38
	$Woodwell_{DC}$	0.03	0.99	1.01	1.89	0.07	0.96	1.77	1.08	0.16	0.92	2.50	0.76
pH	KSSL	0.03	0.96	0.27	9.50	0.14	0.88	0.5	5.13	0.51	0.61	1.72	1.49
(n= 296)	Woodwell	-0.04	0.69	0.82	3.13	0.22	0.85	0.59	4.35	1.34	0.56	2.68	0.96
	$Woodwell_{PDS}$	-0.16	0.82	0.62	4.14	-0.03	0.85	0.56	4.58	-0.73	0.16	2.39	1.07
	$Woodwell_{DC}$	0.04	0.90	0.44	5.83	0.01	0.89	0.46	5.58	0.0	0.84	0.57	4.50

*The performance of $Woodwell_{DC}$ is based on leave-one-out cross validation. The sample size of each property are different because not all predicted soil properties have measured data to evaluate model performance.

Table S6. Performance of NSSC-KSSL predictive models on independent test set (Set III) of the USA soil samples comprising of different combinations of spectra and models (Woodwell, $Woodwell_{PDS}$ and $Woodwell_{DC}$) using the baseline offset transformation.

	Data	MBL				PLSR				Cubist			
		Bias	R^2	RMSE	RPIQ	Bias	R^2	RMSE	RPIQ	Bias	R^2	RMSE	RPIQ
clay	Woodwell	-1.09	0.62	11.31	2.39	2.34	0.67	10.24	2.64	-7.32	0.33	18.75	1.44
(n = 557)	$Woodwell_{PDS}$	-1.02	0.63	10.96	2.46	-0.51	0.67	10.07	2.68	-1.38	0.57	13.49	2.00
	$Woodwell_{DC}$	0.92	0.76	8.50	3.18	0.79	0.76	8.56	3.15	0.57	0.75	8.80	3.07
$CaCO_3$	Woodwell	-0.30	0.93	2.86	0.84	0.20	0.90	3.36	0.71	-1.16	0.92	4.11	0.58
(n = 596)	$Woodwell_{PDS}$	0.06	0.91	3.20	0.75	0.17	0.91	3.32	0.72	0.54	0.74	5.50	0.44
	$Woodwell_{DC}$	0.13	0.93	2.83	0.85	0.15	0.93	2.87	0.84	0.24	0.93	2.82	0.85
OC	Woodwell	-1.30	0.95	3.00	0.76	-1.47	0.95	3.15	0.72	-0.72	0.95	2.80	0.81
(n = 596)	$Woodwell_{PDS}$	0.19	0.96	1.92	1.19	-0.31	0.95	2.63	0.87	0.33	0.94	2.64	0.86
	$Woodwell_{DC}$	0.22	0.94	2.37	0.96	0.34	0.94	2.48	0.92	0.22	0.91	2.73	0.84
pH	Woodwell	0.43	0.65	0.91	2.49	0.48	0.69	0.90	2.52	0.59	0.62	1.16	1.95
(n = 596)	$Woodwell_{PDS}$	0.01	0.75	0.66	3.43	0.03	0.70	0.89	2.54	0.03	0.62	1.07	2.12
	$Woodwell_{DC}$	-0.02	0.82	0.55	4.12	0.00	0.81	0.56	4.04	0.01	0.83	0.54	4.19

*The performance of $Woodwell_{DC}$ is based on leave-one-out cross validation. The sample size of each property are different because not all predicted soil properties have measured data to evaluate model performance.

Table S7. Performance of NSSC-KSSL predictive models on independent test set (Set III) of the USA soil samples comprising of different combinations of spectra and models (Woodwell, $Woodwell_{PDS}$ and $Woodwell_{DC}$) using the first derivative transformation.

	Data	MBL				PLSR				Cubist			
		Bias	R^2	RMSE	RPIQ	Bias	R^2	RMSE	RPIQ	Bias	R^2	RMSE	RPIQ
clay	Woodwell	0.20	0.75	8.81	3.06	-0.37	0.06	12.12	2.23	-17.37	0.10	35.37	0.76
(n = 557)	$Woodwell_{PDS}$	-0.44	0.74	9.01	3.00	1.00	0.65	10.60	2.55	-3.74	0.36	22.07	1.22
	$Woodwell_{DC}$	1.06	0.71	9.43	2.86	1.20	0.65	10.37	2.60	1.15	0.64	10.59	2.55
$CaCO_3$	Woodwell	-0.09	0.92	3.04	0.79	0.35	0.92	3.21	0.75	0.80	0.84	4.41	0.54
(n = 596)	$Woodwell_{PDS}$	-0.22	0.92	2.95	0.81	-0.04	0.92	3.01	0.80	0.32	0.83	4.29	0.56
	$Woodwell_{DC}$	0.13	0.93	2.84	0.85	0.10	0.92	2.89	0.83	0.13	0.88	3.65	0.66
OC	Woodwell	-0.66	0.95	2.57	0.89	-1.00	0.94	2.95	0.77	-2.84	0.75	6.47	0.35
(n = 596)	$Woodwell_{PDS}$	0.02	0.95	2.10	1.09	0.20	0.94	2.51	0.91	-0.16	0.66	7.70	0.30
	$Woodwell_{DC}$	0.16	0.94	2.40	0.95	0.37	0.93	2.74	0.83	0.23	0.92	2.59	0.88
pH	Woodwell	0.02	0.60	0.87	2.60	0.20	0.67	0.81	2.80	0.20	0.37	1.86	1.22
(n = 596)	$Woodwell_{PDS}$	-0.14	0.68	0.76	2.98	-0.07	0.61	0.87	2.60	-0.24	0.18	1.89	1.20
	$Woodwell_{DC}$	-0.04	0.75	0.66	3.43	0.01	0.73	0.68	3.33	-0.02	0.72	0.69	3.28

*The performance of $Woodwell_{DC}$ is based on leave-one-out cross validation. The sample size of each property are different because not all predicted soil properties have measured data to evaluate model performance.

Table S8. Mean correlation coefficients between the primary and secondary spectra with and without PDS transfer for raw and first derivative spectra

	KSSL Primary 1st Der (n=493)	KSSL Primary Raw (n=493)	NEON Val. 1st Der (n =297)	NEON Val. Raw (n=297)
Woodwell	0.92	0.90	0.83	0.80
$Woodwell_{PDS}$	0.95	0.94	0.88	0.86

Table S9. Number of samples detected as outliers using the *F*-ratio approach in Set II and Set III samples using both the baseline offset and first derivative spectra

Sets	Preprocessing	Size	KSSL	Woodwell	$Woodwell_{PDS}$
Set II	Baseline	296	1	2	0
	First Derivative		2	3	2
Set III	Baseline	605	NA	3	1
	First Derivative		NA	3	4

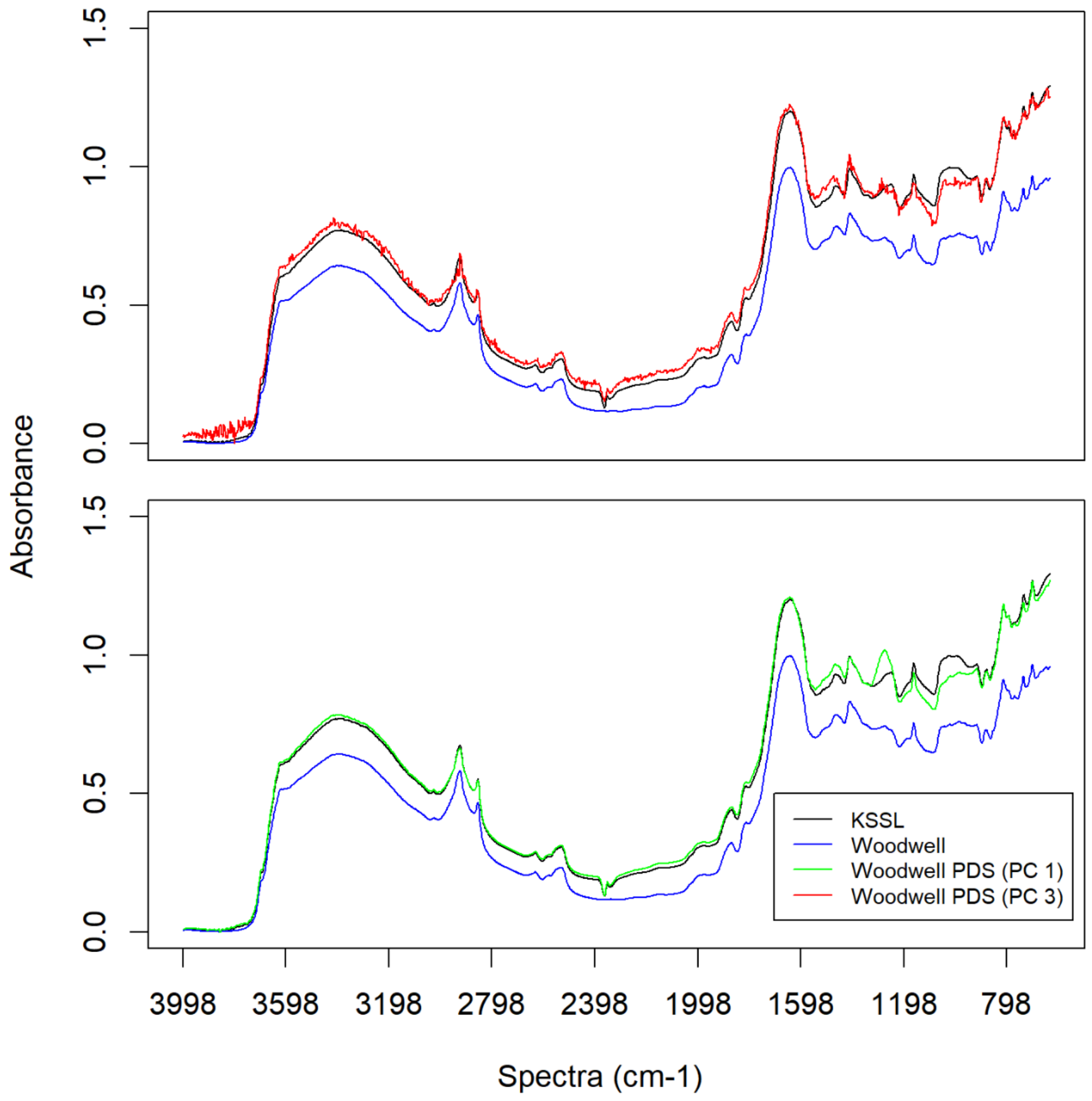

Fig S1. Effect of increasing the number of principal components on the soil spectra with high OC content. The PDS transfer functions were developed using a window size of 3, but with a principal components of 3 (top panel) and 1 (bottom panel). Spectra shown after baseline transformation.

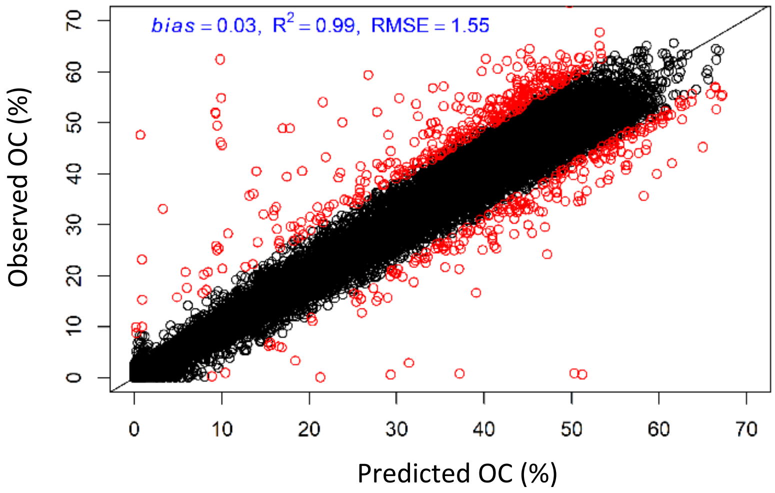

Fig. S2. Outlier detection using the organic carbon (OC) PLSR model the USA NSSC-KSSL spectral library. The red dots are the 1% of samples that have been removed as an outlier, while the black dots are the samples that are retained for developing the PLSR, MBL and Cubist models. The performance statistics in blue represent the statistics of all the samples that have been retained after removing the outliers.

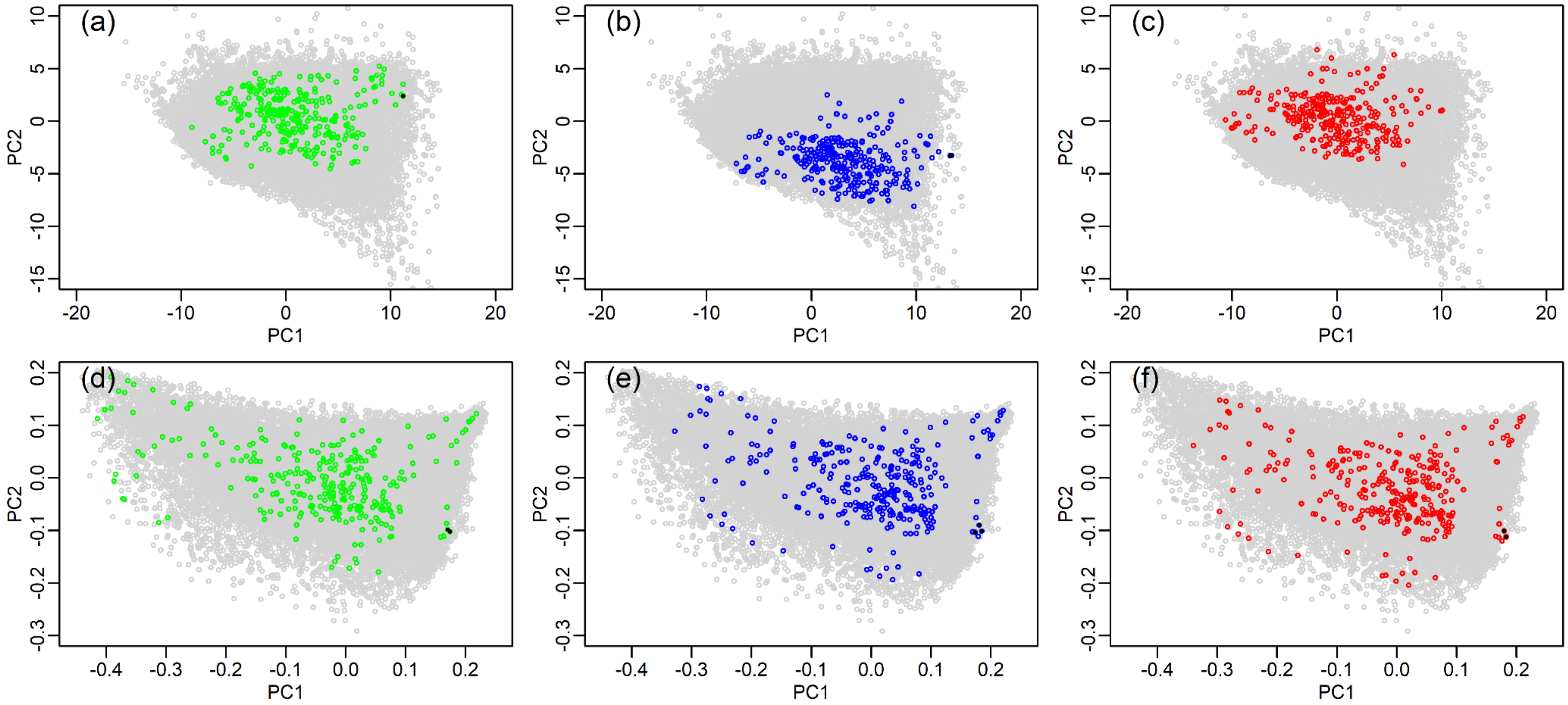

Fig. S3. Detection of outliers (untrustworthy samples) from the validation sets consisting of Set II (NEON) samples. The top and bottom panel figure shows the samples using baseline corrected and first derivative transformation, respectively. The solid black symbols are the samples detected as outliers using the *F-ratio* approach. The background grey circles are calibration samples belonging to the NSSC-KSSL spectral library scanned using the KSSL spectrometer, while the green and blue circles are the samples belonging to Set II and scanned using the KSSL and Woodwell spectrometer, respectively. The red circles are the PDS transfer ($Woodwell_{PDS}$) spectra.

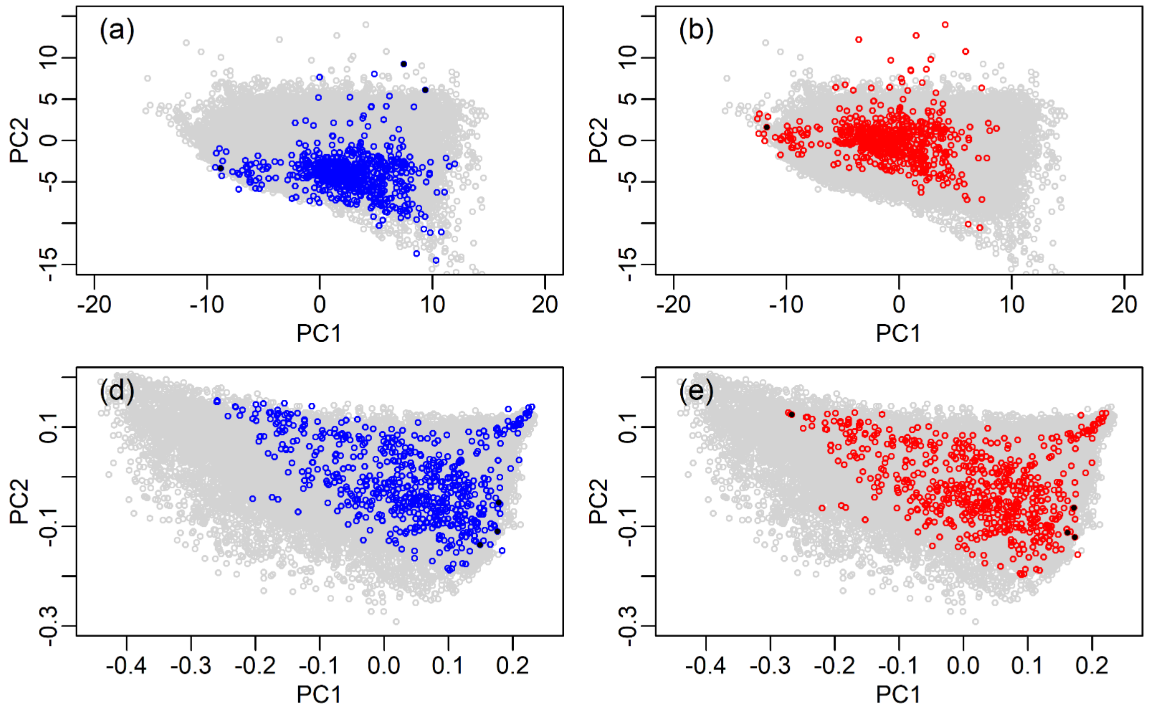

Fig. S4. Detection of outliers (untrustworthy samples) from the validation sets consisting of Set III (LUCAS) samples. The top and bottom panel figure shows the samples using baseline corrected and first derivative transformation, respectively. The solid black symbols are the samples detected as outliers using the *F-ratio* approach. The background grey circles are calibration samples belonging to the NSSC-KSSL spectral library scanned using the KSSL spectrometer, while the blue circles are the samples belonging to Set III and scanned using the Woodwell spectrometer. The red circles are the PDS transfer ($\text{Woodwell}_{\text{PDS}}$) spectra.
